# Pharmacokinetics of doxorubicin following concomitant intravenous administration of olaratumab (IMC‐3G3) to patients with advanced soft tissue sarcoma

**DOI:** 10.1002/cam4.2728

**Published:** 2019-12-10

**Authors:** Victor M. Villalobos, Gary Mo, Mark Agulnik, Seth M. Pollack, Daniel A. Rushing, Arun Singh, Brian A. Van Tine, Rhian McNaughton, Rodney L. Decker, Wei Zhang, Ashwin Shahir, Damien M. Cronier

**Affiliations:** ^1^ University of Colorado Denver Anschutz Medical Campus Aurora CO USA; ^2^ Eli Lilly and Company Indianapolis IN USA; ^3^ Division of Hematology/Oncology Northwestern University Feinberg School of Medicine Chicago IL USA; ^4^ Division of Oncology Fred Hutchinson Cancer Research Center University of Washington Seattle WA USA; ^5^ Simon Cancer Center Indiana University Medical Center Indianapolis IN USA; ^6^ Division of Hematology‐Oncology University of California Los Angeles CA USA; ^7^ Division of Medical Oncology Department of Internal Medicine Siteman Cancer Center St. Louis School of Medicine St. Louis MO USA; ^8^ Eli Lilly and Company Windlesham Surrey UK

**Keywords:** doxorubicin, monoclonal antibody, olaratumab, pharmacokinetics, soft tissue sarcoma

## Abstract

**Background:**

Olaratumab, a fully human monoclonal antibody, selectively binds to human platelet‐derived growth factor receptor alpha and blocks ligand binding. This study assessed the effect of olaratumab on the pharmacokinetics (PK) of doxorubicin and the safety of olaratumab alone and in combination with doxorubicin.

**Methods:**

This open‐label randomized phase 1 trial enrolled 49 patients ages 27 to 83 with metastatic or locally advanced soft tissue sarcoma (STS). Patients participated in 21‐day treatment cycles (up to 8) until they met discontinuation criteria. In cycles 1 and 2, patients received olaratumab (15 mg/kg in Part A, 20 mg/kg in Part B) and doxorubicin (75 mg/m^2^). In cycles 3 through 8, patients continued combination treatment (15 mg/kg olaratumab + doxorubicin). Effect of olaratumab on PK of doxorubicin was determined in patients who received all doses in cycles 1 and 2.

**Results:**

PK properties of doxorubicin administered alone or in combination with olaratumab (15 or 20 mg/kg) were similar for AUC(0‐*t*_last_), AUC(0‐∞), and *C*_max_. PK properties of olaratumab (15 or 20 mg/kg) were also similar when administered alone or in combination with doxorubicin. Three patients died (2 of disease progression and 1 of neutropenic enterocolitis). Fatigue and nausea (>75% of patients) were the most common treatment‐emergent adverse events (TEAEs). Other common TEAEs included musculoskeletal pain, mucositis, constipation, and diarrhea.

**Conclusions:**

Olaratumab at 15 or 20 mg/kg before doxorubicin infusion had no clinically relevant effect on systemic exposure to doxorubicin compared with doxorubicin alone in patients with metastatic or locally advanced STS.

## Graphical abstract

In a phase 1 drug interaction study, the effect of olaratumab on the pharmacokinetics of doxorubicin was determined in patients with advanced soft tissue sarcoma. The pharmacokinetic properties of olaratumab alone or in combination with doxorubicin were similar.

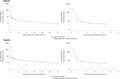

## 1 INTRODUCTION

Soft tissue sarcomas (STS) are a highly variable group of mesenchymal cancers with unmet medical need.^1^ While they account for less than 1% of cancer diagnoses, they encompass over 50 different biological entities with highly variable genetics and behavior.^2^ Drug development has been complex due to high variability of responses in histologic subsets. Until recently, no drugs have been approved for use in all STS, agnostic of subtype, since approval of doxorubicin over 40 years ago. Olaratumab, a fully human monoclonal antibody that selectively binds to human platelet‐derived growth factor receptor alpha (PDGFRα) and blocks ligand binding,^3^ was conditionally approved for treatment of metastatic or incurable STS in combination with doxorubicin.^4^

In a phase 1b/2 trial (I5B‐IE‐JGDG; NCT01185964), olaratumab in combination with doxorubicin met its predefined primary progression‐free survival endpoint and achieved improvement of 11.8 months (HR = 0.46; *P* = .0003) in median overall survival compared to single agent doxorubicin in the metastatic or unresectable setting.^5^ However, olaratumab failed to show clinical efficacy in a phase 3 trial (I5B‐MC‐JGDJ; NCT02451943).

This study evaluated the effect of olaratumab on the pharmacokinetics (PK) of doxorubicin. The secondary objectives were to further characterize the PK and safety profile of olaratumab alone and in combination with doxorubicin. Although the phase 3 study (JGDJ) was unable to confirm efficacy of olaratumab plus doxorubicin in STS patients, the drug‐drug interaction and these safety results provide valuable information for future monoclonal antibody development.

## 2 MATERIALS AND METHODS

### 2.1 Study design and patients

This two‐part, nonrandomized, open‐label study was conducted at seven sites in the United States. Part A evaluated the effect of a 15‐mg/kg dose of olaratumab on the PK of doxorubicin (Figure S1). Part B was added as a protocol amendment after enrollment for Part A was complete to evaluate the effect of a higher dose of olaratumab (20 mg/kg) on the PK of doxorubicin.

Patients were eligible if they were ≥18 years of age, had metastatic or locally advanced STS not amenable to treatment with surgery or curative radiotherapy, an Eastern Cooperative Oncology Group performance status of 0‐2, and an echocardiogram or multigated acquisition scan with an actual left ventricular ejection fraction ≥50%. The trial was conducted in accordance with the principles of the Declaration of Helsinki and Good Clinical Practice guidelines. The protocol was approved by each center's institutional review board, and all patients provided written informed consent.

### 2.2 Treatments and assessments

A treatment cycle was 21 days. Day 1 of cycles 1 through 8 included doxorubicin infusion of 75 mg/m^2^ (Figure S1). Treatment with olaratumab 15 or 20 mg/kg olaratumab IV over approximately 60 minutes began on day 10 of cycle 1 and continued on days 1 and 8 of cycles 2 through 8.

Patients received combination treatment (olaratumab + doxorubicin) for as long as they showed clinical benefit and did not develop unacceptable toxicity, up to 8 cycles. For cycle 9 onwards, patients remained on olaratumab monotherapy. Treatment duration was not fixed; patients continued until they met any criteria for study discontinuation.

### 2.3 Pharmacokinetic bioanalytical method

Plasma concentration of doxorubicin was quantified from venous blood samples collected in cycles 1 and 2. Plasma was analyzed for doxorubicin and doxorubicinol using a validated liquid chromatography with tandem mass spectrometric detection method.

Serum concentrations of olaratumab were quantified from separate venous blood samples. Rich sampling was limited to the first 2 cycles and sparse sampling in cycles 3, 5, and 7. Serum samples were analyzed for olaratumab using an ELISA method. Pharmacokinetic bioanalytical methods are described in Data S1.

### 2.4 Pharmacokinetic analysis

Pharmacokinetic parameters for olaratumab and doxorubicin were determined using noncompartmental methods. Pharmacokinetic parameters of doxorubicin were determined after cycle 1, day 1 (doxorubicin alone) and cycle 2, day 1 (olaratumab + doxorubicin) doses (Table S1).

The same parameters were determined for olaratumab after cycle 1, day 10 (olaratumab alone) and cycle 2, day 1 (olaratumab + doxorubicin) doses of olaratumab, except that the following parameters were calculated in place of AUC(0‐∞), which could not be calculated reliably due to a large number of AUC(0‐∞) determinations with an extrapolation of >20%: AUC from 0 to 168 hours postdose (AUC_0‐168h_) and AUC from 0 to 288 hours postdose (AUC_0‐288h_) were calculated at cycle 1, day 10 and AUC_0‐168h_ and AUC from 0 to 336 hours postdose (AUC_0‐336h_) were calculated at cycle 2, day 1.

Pharmacokinetic analysis methods are further described in the Data S1.

### 2.5 Statistical analysis

The effect of olaratumab on the PK of doxorubicin was determined in all patients who received all study treatment doses in cycles 1 and 2 and had evaluable PK data for the drug‐drug interaction assessment. The doxorubicin PK parameters AUC(0‐*t*_last_), AUC(0‐∞), and *C*_max_ were log transformed and analyzed using a linear mixed‐effects model with treatment (doxorubicin + olaratumab and doxorubicin alone) as a fixed effect and patient as a random effect. The geometric least squares (LS) mean for each treatment and the ratio of geometric LS means (ie, doxorubicin + olaratumab and doxorubicin alone) were calculated along with their 90% confidence intervals (CIs). The analysis was performed separately for Parts A and B.

The relationship between serum concentrations of olaratumab and time‐matched QT interval corrected for heart rate (QTc), which was prespecified to use Fridericia's formula (QTcF), was explored using a linear mixed‐effects model that assessed the effect of olaratumab concentrations on ΔQTcF (defined as QTcF at each time point minus the baseline). The response variable was the time‐matched ΔQTcF value and the independent variable was the time‐matched olaratumab drug concentrations at each time point.

The following linear mixed‐effects model was employed:

$$\begin{array}{cc} \Delta \text{QTcF} & =\text{Intercept}+\text{Slope}*\text{Concentrations}+\text{Random Patient}\\ & \;+\text{Residual Error} \end{array}$$

On the basis of this relationship, the predicted ΔQTcF and its corresponding 90% two‐sided CI was computed at the geometric mean steady‐state *C*_max_ observed in the study.

In addition, a scatterplot of ΔQTcF versus olaratumab concentrations was presented along with the fitted lines.

The mixed‐effects analysis was performed twice: once using data from cycle 1 only and once with data from cycles 1 and 2 combined. Baseline was defined as the average of all cycle 1, day 1 preinfusion readings. It was planned to perform the exposure‐response analyses separately for Parts A and B, but a post hoc analysis was also performed to combine the Parts A and B data into a single scatterplot.

### 2.6 Efficacy analysis

Napoleon plots of duration of exposure and waterfall plots of best percentage change from baseline in tumor size were generated. Within Part A and Part B, plots were generated separately for leiomyosarcoma patients and other patients.

### 2.7 Safety analysis

Safety was analyzed in all patients who entered the study. Treatment‐emergent adverse events (TEAEs) were summarized by treatment and severity. Frequency and percentage of patients who experienced an adverse event (AE) were summarized by treatment, National Cancer Institute Common Terminology Criteria for Adverse Events version 4.02, and Medical Dictionary for Regulatory Activities version 17.0. Serious adverse events (SAEs) were also tabulated.

Adverse events of special interest (AESIs), which consisted of events associated with other agents in a similar class of drugs or that were observed in preclinical evaluation or earlier clinical studies of olaratumab were analyzed. These AESIs were cardiac arrhythmias, cardiac dysfunction, and infusion‐related reaction (IRRs).

## 3 RESULTS

### 3.1 Patient demographics and baseline characteristics

Forty‐nine patients were enrolled, including 25 patients in Part A and 24 patients in Part B (Table 1). Baseline disease characteristics and prior treatment are presented in Table 2.

**Table 1:** Patient demographics

	Part A—75 mg/m^2^ doxorubicin + 15 mg/kg olaratumab (n = 25)	Part B—75 mg/m^2^ doxorubicin + 20 mg/kg olaratumab (n = 24)	Overall (n = 49)
Age (y)			
Mean	56.7	56.1	56.4
SD	12.6	11.2	11.8
Median	57.0	55.0	57.0
Minimum	27	32	27
Maximum	83	72	83
Sex, n (%)			
Male	10 (40.0%)	10 (41.7%)	20 (40.8%)
Female	15 (60.0%)	14 (58.3%)	29 (59.2%)
Ethnicity, n (%)			
Hispanic or Latino	1 (4.0%)	2 (8.3%)	3 (6.1%)
Not Hispanic or Latino	24 (96.0%)	22 (91.7%)	46 (93.9%)
Race, n (%)			
American Indian or Alaska Native	0 (0.0%)	1 (4.2%)	1 (2.0%)
Black or African American	3 (12.0%)	1 (4.2%)	4 (8.2%)
White	22 (88.0%)	22 (91.7%)	44 (89.8%)
Weight (kg)			
Mean	81.6	85.6	83.5
SD	17.3	25.4	21.5
Median	82.1	80.8	82.0
Minimum	46.9	52.5	46.9
Maximum	117.7	156.0	156.0
Height (cm)			
Mean	168.1	171.4	169.7
SD	9.4	11.1	10.3
Median	167.6	171.5	167.6
Minimum	152.4	153.0	152.4
Maximum	185.4	191.8	191.8
Body mass index (kg/m^2^)			
Mean	28.8	28.8	28.8
SD	5.4	6.5	5.9
Median	27.6	28.7	27.7
Minimum	19.1	18.6	18.6
Maximum	43.7	48.0	48.0
Body surface area (m^2^)			
Mean	1.9	2.0	2.0
SD	0.2	0.3	0.3
Median	1.9	1.9	1.9
Minimum	1.4	1.6	1.4
Maximum	2.3	2.7	2.7

**Table 2:** Patient baseline disease characteristics and prior treatment

	Part A—75 mg/m^2^ doxorubicin + 15 mg/kg olaratumab n = 25	Part B—75 mg/m^2^ doxorubicin + 20 mg/kg olaratumab n = 24
ECOG Scale^a^ at screening, n (%)		
0	15 (60.0)	18 (75.0)
1	9 (36.0)	16 (25.0)
2	1 (4.0)	0 (0.0)
Basis of determination, n (%)		
Histopathological	25 (100.0)	19 (79.2)
Cytological	0 (0.0)	5 (20.8)
Pathological disease code, n (%)		
Leiomyosarcoma, pleomorphic	1 (4.0)	2 (8.3)
Leiomyosarcoma, poorly differentiated, back	0 (0.0)	1 (4.2)
Liposarcoma, CNS	0 (0.0)	1 (4.2)
Rhabdomyosarcoma	1 (4.0)	0 (0.0)
Sarcoma, angiosarcoma, heart	1 (4.0)	0 (0.0)
Sarcoma, clear cell	1 (4.0)	0 (0.0)
Sarcoma, fibrosarcoma	1 (4.0)	0 (0.0)
Sarcoma, leiomyosarcoma, NOS	4 (16.0)	3 (12.5)
Sarcoma, leiomyosarcoma, abdomen (Non‐Gist)	2 (8.0)	1 (4.2)
Sarcoma, leiomyosarcoma, angiosarcoma	2 (8.0)	0 (0.0)
Sarcoma, leiomyosarcoma, femur^b^, ^c^	1 (4.0)	1 (4.2)
Sarcoma, leiomyosarcoma, uterine	2 (8.0)	2 (8.3)
Sarcoma, liposarcoma	4 (16.0)	4 (16.7)
Sarcoma, malignant fibrous histiocytoma	1 (4.0)	0 (0.0)
Sarcoma, myxofibrosarcoma	1 (4.0)	2 (8.3)
Sarcoma, myxoliposarcoma	1 (4.0)	1 (4.2)
Sarcoma, neurofibrosarcoma	0 (0.0)	1 (4.2)
Sarcoma, NOS	2 (8.0)	4 (16.7)
Sarcoma, stromal, endometrial	0 (0.0)	1 (4.2)
Disease stage, n (%)		
Stage I	1 (4.0)	0 (0.0)
Stage IA	0 (0.0)	1 (4.2)
Stage IB	0 (0.0)	1 (4.2)
Stage IC	0 (0.0)	0 (0.0)
Stage II	0 (0.0)	0 (0.0)
Stage IIA	1 (4.0)	0 (0.0)
Stage IIB	4 (16.0)	2 (8.3)
Stage IIC	0 (0.0)	0 (0.0)
Stage III	7 (28.0)	6 (25.0)
Stage IV	5 (20.0)	5 (20.8)
Metastatic	7 (28.0)	7 (29.2)
Local	0 (0.0)	2 (8.3)
Prior treatment or surgery, n (%)		
None	1 (4.0)	1 (4.2)
Diagnostic surgery only	2 (8.0)	1 (4.2)
Systemic chemotherapy only	0 (0.0)	1 (4.2)
Diagnostic surgery and systemic chemotherapy	1 (4.0)	2 (8.3)
Diagnostic surgery and radiotherapy	0 (0.0)	1 (4.2)
Palliative and/or curative surgery with or without systemic chemotherapy and/or radiation therapy	21 (84.0)	18 (75.0)

Abbreviations: CNS, central nervous system; ECOG, Eastern Cooperative Oncology Group; NOS, not otherwise specified.

a ECOG Performance Status: 0 = Fully active, able to carry on all pre‐disease performance without restriction; 1 = Restricted in physically strenuous activity but ambulatory and able to carry out work of a light or sedentary nature, eg, light house work, office work; 2 = Ambulatory and capable of all self‐care but unable to carry out any work activities; up and about more than 50% of waking hours.

b Investigator confirmed that in Part A diagnosis of ‘sarcoma, leiomyosarcoma, femur’ was not a bone tumor but a soft tissue leiomyosarcoma.

c Investigator confirmed that in Part B diagnosis of ‘sarcoma, leiomyosarcoma, femur’ was not a bone tumor but a sarcoma in the patient's inguinal area that later spread to the lungs.

### 3.2 Doxorubicin pharmacokinetics

**Figure 1:**
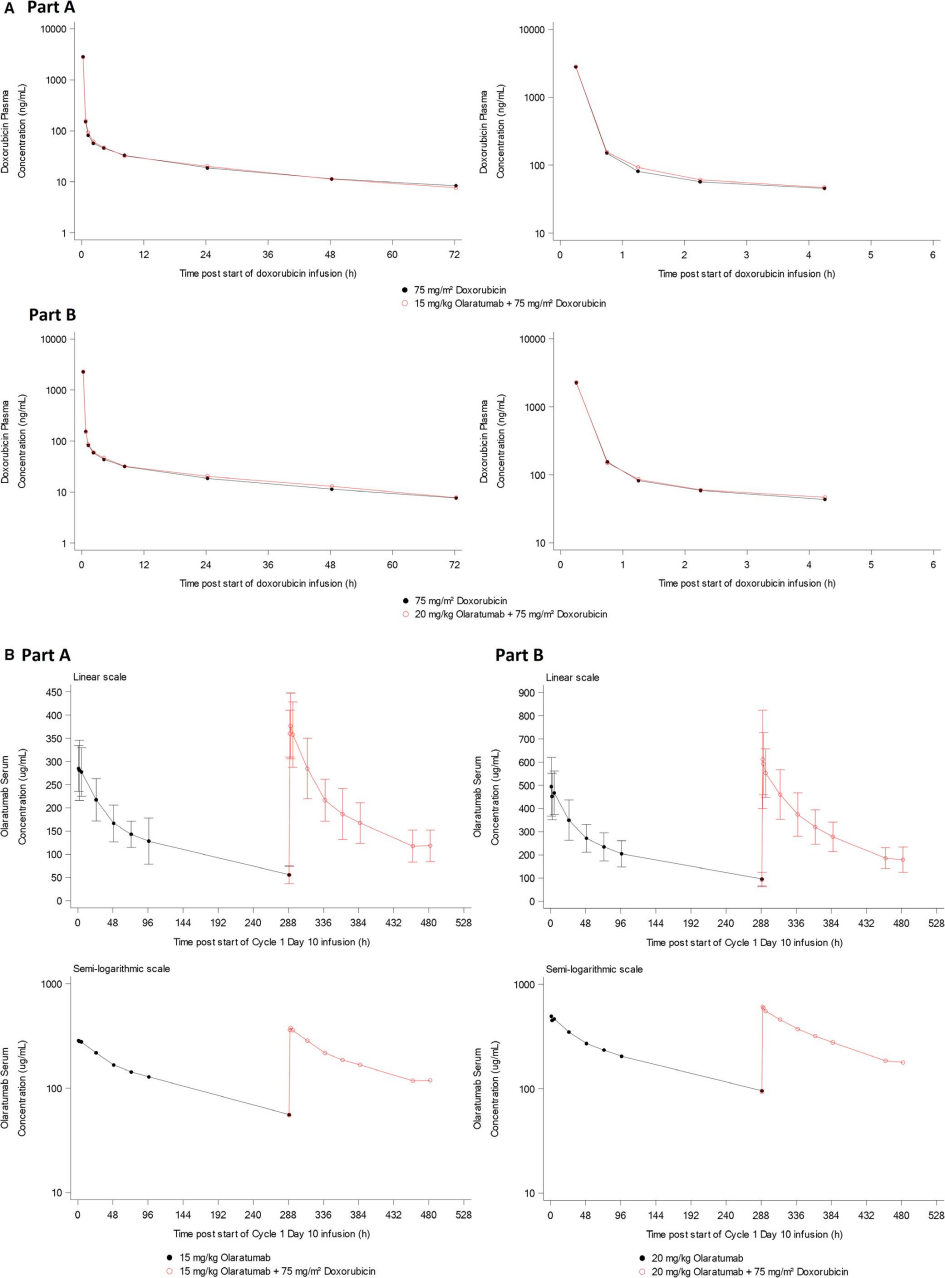
A, Arithmetic mean plasma concentration profiles of doxorubicin following intravenous administration of doxorubicin with or without 15 mg/kg (Part A) or 20 mg/kg (Part B) olaratumab. B, Arithmetic mean (±SD) serum concentration profiles of olaratumab following intravenous administration of 15 mg/kg (Part A) or 20 mg/kg (Part B) olaratumab with or without doxorubicin. B, Arithmetic mean (±SD) serum concentration profiles of olaratumab following intravenous administration of 15 mg/kg (Part A) or 20 mg/kg (Part B) olaratumab with or without doxorubicin

Mean doxorubicin concentration‐time profiles following a 15‐minute infusion of 75 mg/m^2^ doxorubicin alone or after a 1‐hour infusion of 15 mg/kg olaratumab are presented in Figure 1A. Mean PK profiles of doxorubicin administered alone or in combination with olaratumab were similar in Parts A and B, and the PK parameters of doxorubicin showed no marked difference (Table 3). Analysis of relevant PK parameters (Table 4) showed no statistically significant effect of olaratumab on doxorubicin PK as the 90% CIs for the ratios of geometric LS means for AUC(0‐∞) and AUC(0‐*t*_last_) were within the standard no‐effect boundary (0.8, 1.25). The lower limit of the 90% CI for *C*_max_ was slightly outside the no‐effect boundary, but the ratio was still close to unity.

**Table 3:** Pharmacokinetic parameters of 75 mg/m^2^ doxorubicin alone or with olaratumab

Parameter	Part A—75 mg/m^2^ doxorubicin + 15 mg/kg olaratumab		Part B—75 mg/m^2^ doxorubicin + 20 mg/kg olaratumab	
	Geometric mean (CV%)			
	Doxorubicin only (N = 23) (cycle 1, day 1)	Doxorubicin + olaratumab (N = 22) (cycle 2, day 1)	Doxorubicin only (N = 24) (cycle 1, day 1)	Doxorubicin + olaratumab (N = 21) (cycle 2, day 1)
AUC(0‐*t*_last)_ (ng∙h/mL)	2240 (25)^a^	2240 (29)^b^	2060 (23)^a^	2180 (22)
AUC(0‐∞) (ng∙h/mL)	2580 (24)	2570 (28)	2400 (21)	2470 (20)
%AUC(0‐*t*_last)_	13.2 (27)	11.9 (22)	12.7 (32)	11.4 (28)
*C*_max_ (ng/mL)	2570 (47)	2330 (68)	2060 (53)	2070 (60)
CL (L/h)	55.6 (30)	56.8 (35)	61.6 (29)	59.6 (33)
*t*_1/2_ (h)^c^	36.4 (22.4‐56.1)^e^	36.1 (18.5‐48.1)^f^	35.5 (20.6‐83.2)^g^	33.6 (21.1‐43.8)^h^
*t*_max_ (h)^d^	0.30 (0.25‐0.58)	0.31 (0.25‐0. 58)	0.33 (0.25‐0.67)	0.33 (0.25‐0. 55)
*V*_z_ (L)	2920 (25)	2960 (26)	3150 (34)	2890 (32)
*V*_ss_ (L)	1750 (34)	1780 (37)	2000 (36)	1880 (32)

Abbreviations: AUC(0‐∞), area under the concentration‐time curve (AUC) from zero to infinity; AUC(0‐*t*_last_), AUC from zero to time *t*, where t is the last time point with a measurable concentration; %AUC(*t*_last_‐∞), fraction of AUC(0‐∞) extrapolated; CL, total body clearance of drug calculated after intravenous (IV) administration; *C*_max_, maximum observed drug concentration; CV%, coefficient of variation; N, number of patients studied; *t*_1/2_, half‐life associated with the terminal rate constant in noncompartmental analysis; *t*_max_, time of *C*_max_; *V*_z_, volume of distribution during the terminal phase; *V*_ss_, volume of distribution at steady state following IV administration.

a N = 22.

b N = 21.

c Geometric mean (range).

d Median (range). Times are relative to start of 15‐minute IV infusion of doxorubicin.

e Sixteen individual *t*_1/2_ estimates used were calculated over a period of less than twice resultant half‐life; result should be interpreted with caution.

f Fifteen individual *t*_1/2_ estimates used were calculated over a period of less than twice resultant half‐life; result should be interpreted with caution.

g Fourteen individual *t*_1/2_ estimates used were calculated over a period of less than twice resultant half‐life; result should be interpreted with caution.

h Seven individual *t*_1/2_ estimates used were calculated over a period of less than twice resultant half‐life; result should be interpreted with caution.

**Table 4:** Statistical analysis of the effect of olaratumab on the pharmacokinetic parameters of doxorubicin

Parameter	Treatment	Part A—75 mg/m^2^ doxorubicin + 15 mg/kg olaratumab			Part B—75 mg/m^2^ doxorubicin + 20 mg/kg olaratumab		
		N	Geometric LS means	Ratio of geometric LS means (doxorubicin + olaratumab:doxorubicin) (90% confidence interval)	N	Geometric LS means	Ratio of geometric LS means (doxorubicin + olaratumab:doxorubicin) (90% confidence interval)
AUC(0‐∞) (ng h/mL)	Doxorubicin only	21	2489	1.04 (0.964, 1.13)	21	2397	1.03 (0.957, 1.11)
	Doxorubicin + olaratumab	21	2598		21	2468	
AUC(0‐*t*_last_) (ng h/mL)	Doxorubicin only	19	2175	1.06 (0.969, 1.16)	19	2054	1.05 (0.962, 1.15)
	Doxorubicin + olaratumab	19	2308		19	2161	
*C*_max_ (ng/mL)	Doxorubicin only	21	2522	0.944 (0.770, 1.16)	21	2009	1.03 (0.801, 1.33)
	Doxorubicin + olaratumab	21	2380		21	2070	

Abbreviations: AUC(0‐∞), area under the concentration‐time curve (AUC) from zero to infinity; AUC(0‐*t*_last_), AUC from zero to time *t*, where *t* is the last time point with a measurable concentration; *C*_max_, maximum observed drug concentration; LS, least squares; N, number of patients studied; PK, pharmacokinetic.

Model: Log(PK) = patient + treatment +random error, where patient is fitted as a random effect.

### 3.3 Olaratumab pharmacokinetics

Mean serum concentration‐time profiles of olaratumab following a 1‐hour infusion of olaratumab at 15 mg/kg alone or in combination with 75 mg/m^2^ doxorubicin are shown in Figure 1B. After the first infusion of olaratumab alone on day 10 of cycle 1, mean *C*_max_ of 292 μg/mL was achieved 1 hour after infusion, then declined slowly. Olaratumab serum levels remained quantifiable until the next infusion (approximately 288 hours). The PK profile of olaratumab was similar after infusion of 15 mg/kg olaratumab + 75 mg/m^2^ doxorubicin on day 1 of cycle 2 (Figure 1B). Mean *t*_max_ was slightly later, approximately 2.8 hours after start of the infusion. The mean *C*_max_ of olaratumab in cycle 2 was higher than the *C*_max_ in cycle 1 due to residual olaratumab serum concentrations at the time of cycle 2 infusion (preinfusion serum concentration was 55.6 μg/mL on day 1 of cycle 2). Mean olaratumab *t*_1/2_ was 121 hours. Comparison of estimated olaratumab CL, *t*_1/2_, *t*_max_, *V*_z_, and *V*_ss_ alone or in combination with 75 mg/m^2^ doxorubicin showed no marked difference in PK parameters between treatments (Table 5). Similar to Part A, there was no remarkable difference in the time profile when 20 mg/kg olaratumab was administered alone or in combination with doxorubicin. Differences in concentration values, such as *C*_max_ and AUC, are due to accumulation of olaratumab in subsequent infusions. Because of the accumulation of olaratumab between monotherapy dose and combination dose, a comparison of concentration values is inappropriate.

**Table 5:** Pharmacokinetic parameters of olaratumab alone or with doxorubicin

Parameter	Part A—15 mg/kg olaratumab + 75 mg/m^2^ doxorubicin		Part B—20 mg/kg olaratumab + 75 mg/m^2^ doxorubicin	
	Geometric mean (CV%)			
	Olaratumab only (cycle 1, day 10) (N = 23)	Olaratumab + doxorubicin (cycle 2, day 1) (N = 24)	Olaratumab only (cycle 1, day 10) (N = 23)	Olaratumab + doxorubicin (cycle 2, day 1) (N = 23)
AUC (0‐*t*_last_) (μg h/mL)^a^	32 800 (21)	32 000 (21)^b^	53 800 (24)^c^	54 100 (20)^c^
AUC_0‐168h_ (μg h/mL)	24 600 (21)	32 200 (21)	40 100 (22)	52 900 (22)
AUC_0‐288h_ (μg h/mL)	32 900 (22)	NC	53 800 (24)	NC
AUC_0‐336h_ (μg h/mL)	NC	44 200 (23)	NC	71 900 (23)
*C*_max_ (μg/mL)	292 (19)	386 (16)	512 (21)	634 (26)
CL (L/h)	0.0261 (35)	0.0230 (32)	0.0220 (36)	0.0193 (39)
*t*_1/2_ (h)^d^	154 (80.3‐214)^e^	121 (55.9‐195)^f^	163 (67.4‐241)^g^	117 (72.8‐264)^h^
*t*_max_ (h)^i^	2.00 (1.00‐23.17)	2.79 (1.80‐6.43)	1.67 (0.05‐24.10)	3.50 (1.00‐6.97)
*V*_z_ (L)	5.79 (26)	4.02 (30)	5.15 (28)	3.27 (29)
*V*_ss_ (L)	5.56 (24)	3.95 (27)	4.92 (27)	3.23 (27)

Abbreviations: AUC_0‐168h_, area under the concentration‐time curve (AUC) from 0 to 168 h postdose; AUC_0‐288h_, AUC from 0 to 288 h postdose; AUC_0‐336h_, AUC from 0 to 336 h postdose; AUC(0‐*t*_last_), AUC from zero to time *t*, where *t* is the last time point with a measurable concentration; CL, total body clearance of drug calculated after intravenous (IV) administration; *C*_max_, maximum observed drug concentration; CV%, coefficient of variation; N, number of patients studied; NC, not calculated; *t*_1/2_, half‐life associated with the terminal rate constant in noncompartmental analysis; *t*_max_, time of *C*_max_; *V*_z_,volume of distribution during the terminal phase; *V*_ss_, volume of distribution at steady state following IV administration.

a AUC(0‐*t*_last_) values are not directly comparable between cycles 1 and 2 because they were calculated over different periods of time.

b N = 23.

c N = 22.

d Geometric mean (range).

e Nineteen individual *t*_1/2_ estimates used were calculated over a period of less than twice the resultant half‐life; result should be interpreted with caution.

f All of the 24 individual *t*_1/2_ estimates used were calculated over a period of less than twice the resultant half‐life; result should be interpreted with caution.

g Twenty‐one individual *t*_1/2_ estimates used were calculated over a period of less than twice the resultant half‐life; result should be interpreted with caution.

h Twenty‐two individual *t*_1/2_ estimates used were calculated over a period of less than twice the resultant half‐life; result should be interpreted with caution.

i Median (range). Times are relative to the start of 60‐min IV infusion of olaratumab.

### 3.4 Efficacy

**Figure 2:**
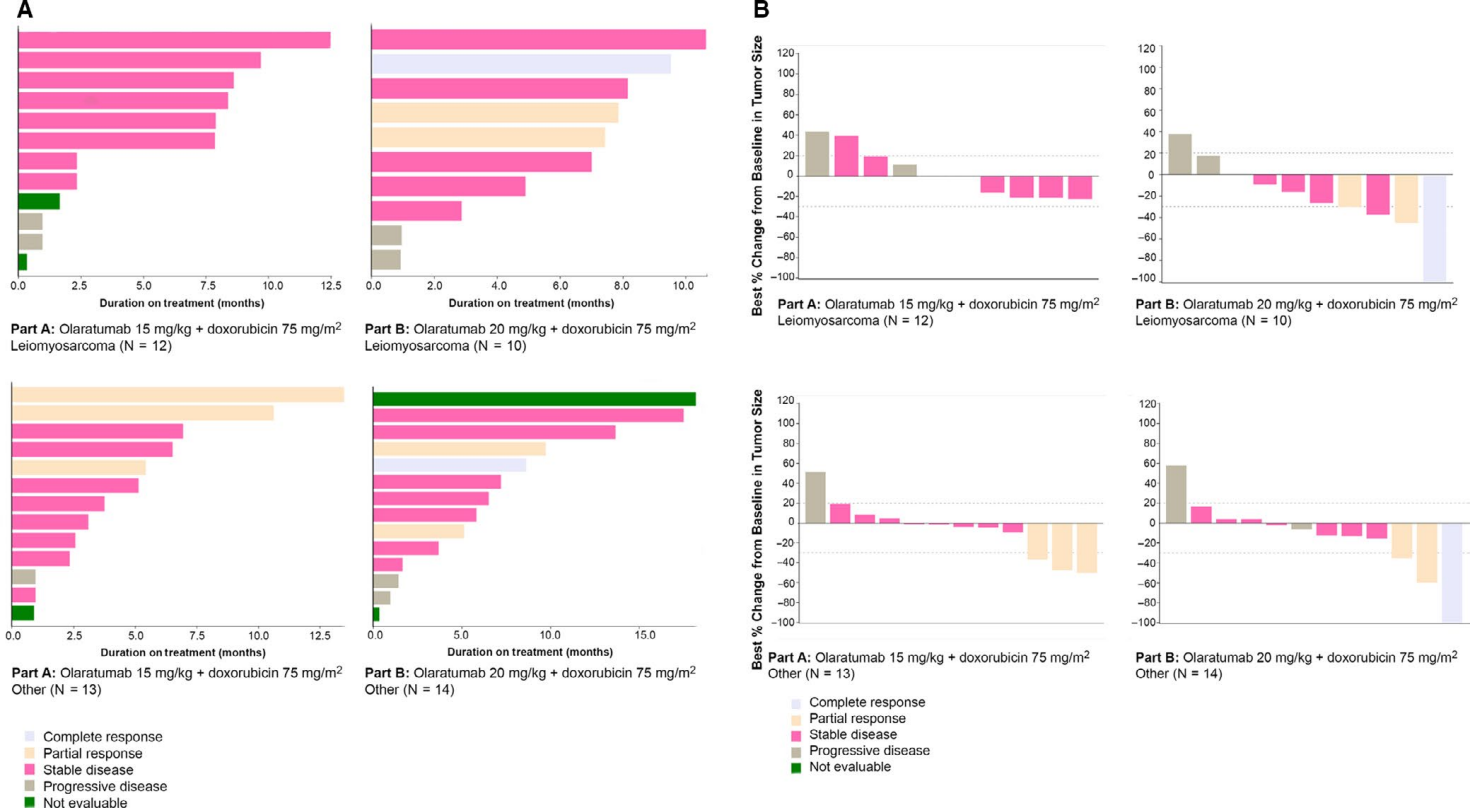
A, Duration of exposure to treatment (safety population). Part A: Olaratumab 15 mg/kg + doxorubicin 75 mg/mg^2^; Part B: Olaratumab 20 mg/kg + doxorubicin 75 mg/mg^2^. The Napoleon plot shows the number of months on treatment and the best overall response based on RECIST Version 1.1 for individual patients in Part A (75 mg/m2 doxorubicin + 15 mg/kg olaratumab) and Part B (75 mg/m2 doxorubicin + 20 mg/kg Olaratumab) portions of the study. Each bar represents one patient. B, Waterfall plots of best percentage change in tumor size (safety population)

Patient response to treatment was assessed using duration of treatment and tumor size according to Response Evaluation Criteria in Solid Tumors (RECIST) criteria. Duration of treatment was examined for 49 patients and was separated based on olaratumab dosing in the two parts of the study and patient tumor subtype of leiomyosarcoma versus other STS subtypes (Figure 2A). In Part A, there were 12 patients with leiomyosarcoma and 13 patients with other STS tumors. In Part B there were 10 patients with leiomyosarcoma and 14 patients with other STS tumors. Tumor response, as per RECIST criteria, for each patient was also incorporated into the figure. Tumor response is presented in Figure 2B, and the same patient grouping was used as for the treatment duration assessment.

### 3.5 Safety results

The most common TEAEs reported in Part A were fatigue and nausea (>50% of patients), and anemia, musculoskeletal pain, neutropenia, thrombocytopenia, mucositis, constipation, and cough were also reported by more than one‐third of patients (Table S2).

The most common TEAEs reported in Part B were fatigue and nausea (>75% of patients). Other common treatment‐related TEAEs in Part B (reported by at least one‐third of patients) included musculoskeletal pain, mucositis, constipation, diarrhea, alopecia, neutropenia, anemia, cough, decreased appetite, dysgeusia, and vomiting. There were no notable differences in AEs experienced by patients in Part A and Part B.

Three patients died during the study: 1 patient in Part A died after cycle 12, day 8 due to disease progression; 1 patient in Part B died after cycle 10, day 8 due to disease progression; and a second patient in Part B died of an SAE of neutropenic enterocolitis (typhlitis). Three patients (6.1%), all in Part A, discontinued the study due to an AE (Table S2).

**Figure 3:**
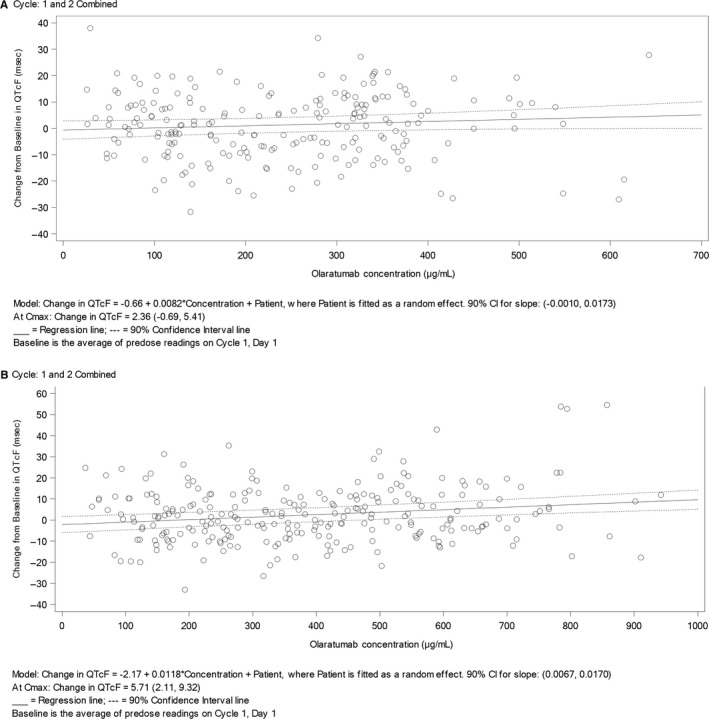
Scatterplot of changes from baseline (cycle 1, day 1 predose) in QTcF values versus serum concentrations of olaratumab following (A) 15 mg/kg dose of olaratumab or (B) 20 mg/kg dose of olaratumab changes from baseline (cycle 1, day 1 predose) in QTcF values versus serum concentrations of olaratumab following a 15 mg/kg dose of olaratumab and 20 mg/kg dose of olaratumab

Importantly, the overall rate of AESIs of IRR, cardiac arrhythmias, and cardiac dysfunction was similar between patients in Part A and Part B (Table S2). IRRs were reported with olaratumab during Part A (n = 2; 8%); no IRRs were reported in Part B. There were no fatal events of IRR. No clinically significant relationship between olaratumab exposure and change in QTcF was seen as the upper bound of 90% CI at *C*_max_ did not exceed 10 ms (Figure 3A,B). No patients showed a QTcF value >480 ms, and no patients had an increase from baseline in QTcF >60 ms following administration of either 15 or 20 mg/kg of olaratumab.

## 4 DISCUSSION

The PK profiles of doxorubicin observed following IV infusion of 75 mg/m^2^ doxorubicin in metastatic or locally advanced STS patients were in accordance with expectations for this population based on previous studies.^6^ Infusion of 15 mg/kg or 20 mg/kg olaratumab before a 75 mg/m^2^ doxorubicin infusion did not have a clinically relevant effect on the exposure to doxorubicin. The difference in estimated AUC(0‐*t*_last_) and AUC(0‐∞) of doxorubicin with or without olaratumab was not statistically significant. Although the 90% CIs for *C*_max_ were slightly outside the no‐effect boundary, the difference was too small to warrant any dose adjustments and therefore has no major clinical implication on the treatment strategy for olaratumab in combination with doxorubicin in STS patients.

The PK properties of olaratumab in this study were consistent with those previous reported.^5^, ^7^ Assessment of the effects of doxorubicin on the PK of olaratumab must be done with care as the accumulation of olaratumab following subsequent infusions affects PK parameters, such as AUCs, *C*_max_, and *C*_min_. Therefore, AUC, *C*_max_ and *C*_min_ values are not appropriate for evaluation of potential effect of doxorubicin on PK of olaratumab. However, PK parameters such as CL, *t*_1/2_, *t*_max_, *V*_z_, and *V*_ss_ were not affected by accumulation given the previously reported dose‐proportionality of olaratumab.^7^ The estimates of these PK parameters for olaratumab were similar when olaratumab was administered alone or in combination with doxorubicin. These findings are consistent with our current understanding of the mechanisms in the elimination of monoclonal antibodies.^8^

We assessed efficacy using treatment duration and change in tumor size. Several patients continued to have stable disease while receiving olaratumab alone after finishing their doxorubicin dosing. Based on phase 3 results, it is unclear whether this is a residual benefit from doxorubicin dosing or whether this may represent continued disease stabilization from continued olaratumab. The effects of olaratumab (in combination with doxorubicin or as monotherapy) in the setting of metastatic sarcomas should be interpreted with caution due to the lack of efficacy during the Phase 3 study.^9^

No notable safety concerns were identified. The most common toxicities experienced in this study included gastrointestinal and hematological events, which is consistent with known toxicities associated with doxorubicin.^10^ AEs were monitorable and predominantly Grade ≤2. Overall, the rate of AESIs of cardiac arrhythmias and cardiac dysfunction was similar between patients in Part A and Part B, which is consistent with the known safety profile of olaratumab in combination with doxorubicin.

As with other monoclonal antibodies,^11^ IRRs were reported in this study. Overall indicence of IRRs was low, consistent with previous study of olaratumab treatment in patients with STS.^5^ There was no evidence of QT prolongation following administration of either 15 or 20 mg/kg olaratumab in patients with metastatic or locally advanced STS.

### 4.1 Conclusions

Intravenous infusion of 15 or 20 mg/kg olaratumab before a 75‐mg/m^2^ doxorubicin infusion did not have a clinically relevant effect on systemic exposure to doxorubicin compared with infusion of doxorubicin alone in patients with metastatic or locally advanced STS. The combination of olaratumab and doxorubicin has an acceptable and monitorable safety profile.

## CONFLICT OF INTEREST

VM Villalobos is an advisory board member for Eli Lilly and Company. G. Mo, R. McNaughton, RL Decker, W. Zhang, and A. Shahir are employees and shareholders of Eli Lilly and Company. M Agulnik reports personal fees from Bayer, Bristol‐Myers Squibb, Eli Lilly and Company, Immune Design, Janssen Pharmaceutica, and Novartis. SM Pollack reports personal fees from Bayer, Blueprint Medicines, Daiichi Sankyo, Eisai, Eli Lilly and Company, PureTech, Seattle Genetics, and Tempus. DA Rushing is an advisory board member for Bayer, Eisai Pharmaceuticals, and Eli Lilly and Company and reports personal fees from each. A. Singh is an advisory board member for Eli Lilly and Company and serves on the speaker's bureau for Novartis. He reports personal fees from Blueprint Medicines, Eli Lilly and Company, and OncLive; grants from Blueprint Medicines, Bristol Myers Squibb, Deciphera, Eli Lilly and Company, and Nanocarrier; and owns stock in Certis Oncology Solutions. BA Van Tine reports personal fees from Adaptimmune Therapeutics, Caris Life Sciences, CytRx Corporation, Daiichi Sankyo, Eli Lilly and Company, Epizyme, Immune Design, Janssen, and Plexxicon, and grants from, Merck, Pfizer, and Tracon Pharmaceuticals. D. Cronier owns stock in Eli Lilly and Company.

## AUTHOR CONTRIBUTIONS

Victor M. Villalobos, Mark Agulnik, Seth M. Pollack, Daniel A. Rushing, Arun Singh, Brian A. Van Tine: Acquisition, analysis and interpretation of data, review and revision of manuscript. Gary Mo: Conceptualization, design, methodology, analysis and interpretation of data, and writing, review, and revision of manuscript. Rhian McNaughton, Damien Cronier: Analysis and interpretation of data, review and revision of manuscript. Rodney L. Decker: Acquisition of data, analysis and interpretation of data, review and revision of manuscript. Wei Zhang: Interpretation of data and drafting and critical revision of manuscript. Ashwin Shahir: Conceptualization, design, methodology, analysis and interpretation of data, and drafting of manuscript. Acquisition of data: VM Villalobos, G. Mo, M. Agulnik, SM Pollack, DA Rushing, A. Singh, BA Van Tine, R. Decker.

## Data Availability

Lilly provides access to all individual participant data collected during the trial, after anonymization, with the exception of pharmacokinetic or genetic data. Data are available to request 6 months after the indication studied has been approved in the US and EU and after primary publication acceptance, whichever is later. No expiration date of data requests is currently set once data are made available. Access is provided after a proposal has been approved by an independent review committee identified for this purpose and after receipt of a signed data sharing agreement. Data and documents, including the study protocol, statistical analysis plan, clinical study report, blank or annotated case report forms, will be provided in a secure data sharing environment. For details on submitting a request, see the instructions provided at http://www.vivli.org.
