# Supplementary material for: Pharmacokinetics of doxorubicin following concomitant intravenous administration of olaratumab (IMC‐3G3) to patients with advanced soft tissue sarcoma
Source: Cancer Med. 2019 Dec 10;9(3):882–93. doi: 10.1002/cam4.2728 (PMC6997100; doi:10.1002/cam4.2728)
Supplement: Supplementary file 1 [file CAM4-9-882-s001.pdf]

Pharmacokinetic Bioanalytical Method

Plasma concentration of doxorubicin was quantified from venous blood samples collected frequently in cycles 1 and 2. Plasma was analyzed for doxorubicin and doxorubicinol using a validated liquid chromatography with tandem mass spectrometric detection method at Intertek Pharmaceutical Services (El Dorado Hills and San Diego, California, USA). The LLOQ (lower limit of quantification) was 5.00 ng/mL, and the upper limit of quantification was 1000 ng/mL for both analytes. Samples above the upper limit of quantification were diluted to yield results within the calibrated range. The inter-assay accuracy (% relative error) during validation ranged from -4.14% to -1.46% (-4.18% at the LLOQ) for doxorubicin and -1.60% to -0.11% (-6.57% at the LLOQ) for doxorubicinol. The inter-assay precision (% relative SD) during validation ranged from 2.45% to 2.92% (7.90% at the LLOQ) for doxorubicin and 2.72% to 3.65% (5.19% at the LLOQ) for doxorubicinol. For doxorubicin, low- and high-quality control samples were stable for 175 days, the 6000.00 ng/mL and 20,000.00 ng/mL dilution quality control samples were stable for 127 days, and the 30,000.00 ng/mL dilution quality control samples were stable for 19 days at approximately -20°C. Doxorubicinol was stable for up to 175 days when stored at approximately -20°C. Doxorubicin was stable for up to 389 days, and doxorubicinol was stable for up to 456 days at approximately -70°C. Serum concentrations of olaratumab were quantified from separate venous blood samples collected from patients in the study. Rich and/or sparse sampling was conducted according to study protocol, where rich sampling was limited to the first 2 cycles and sparse sampling in cycles 3, 5, and 7. Serum samples were analyzed for

olaratumab using an ELISA method at ICON Laboratory Services, Inc. (Whitesboro, NY, USA). Olaratumab was stable for up to 1052 days when stored at approximately -70°C.

Pharmacokinetic Analysis

Pharmacokinetic parameters for olaratumab and doxorubicin were determined using noncompartmental methods using Phoenix WinNonlin (Version 6.4., Cetara, Princeton, NY). Pharmacokinetic parameters of doxorubicin were determined after cycle 1, day 1 (doxorubicin alone) and cycle 2, day 1 (olaratumab + doxorubicin) doses (Supplemental Table 1).

The same parameters were determined for olaratumab after cycle 1, day 10 (olaratumab alone) and cycle 2, day 1 (olaratumab + doxorubicin) doses of olaratumab, except that the following parameters were calculated in place of AUC(0-∞), which could not be calculated reliably due to a large number of AUC(0-∞) determinations with an extrapolation of >20%: AUC from 0 to 168 hours postdose ($AUC_{0\text{-}168h}$) and AUC from 0 to 288 hours postdose ($AUC_{0\text{-}288h}$) were calculated at cycle 1, day 10 and $AUC_{0\text{-}168h}$ and AUC from 0 to 336 hours postdose ($AUC_{0\text{-}336h}$) were calculated at cycle 2, day 1. Actual sampling times relative to the start of the infusion of olaratumab (for olaratumab PK parameters) or doxorubicin (for doxorubicin PK parameters) were used in the analyses of individual PK parameters, with the exception of pre-first-dose sampling times on the first dosing day, which were set to zero. Plasma or serum concentration values below the LLOQ were excluded from the analysis. For graphical assessment, average concentrations at any individual time point were only calculated if at least two thirds of the individual data were above the LLOQ.

Supplemental Figure. 1. Study design.

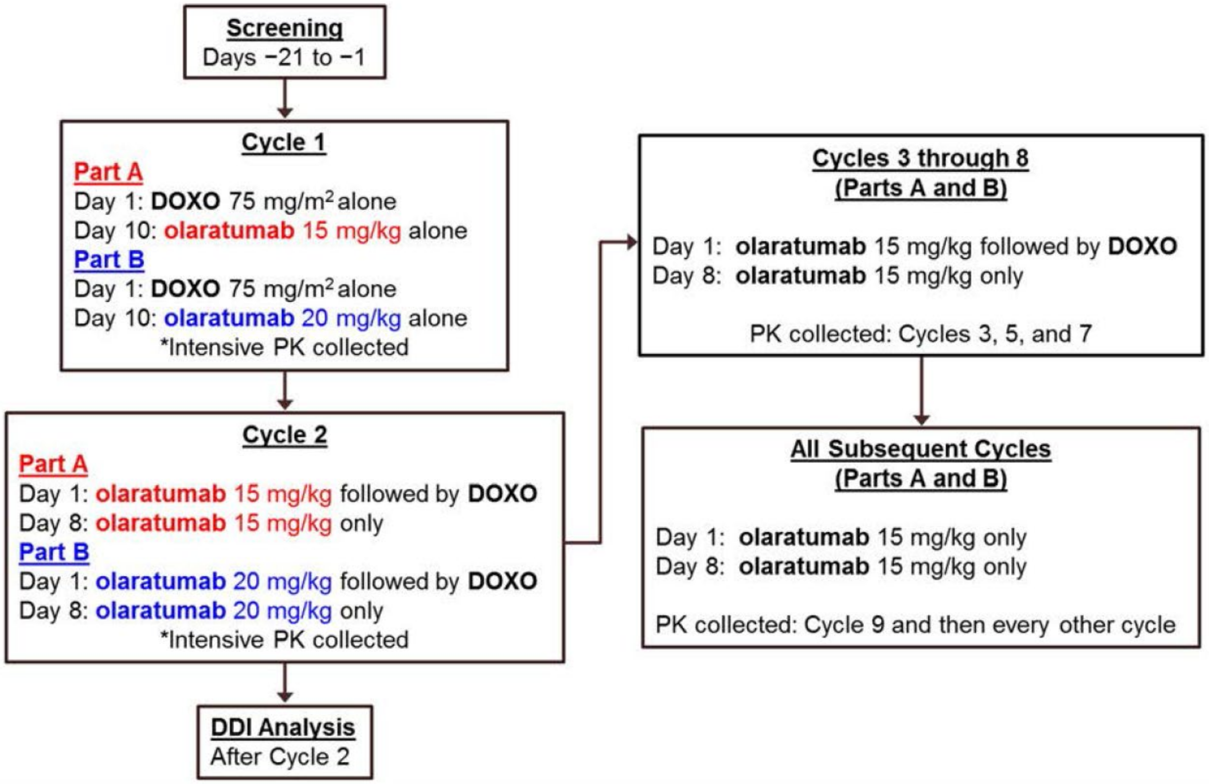

***Cycles 1 and 2 PK Sampling Times:**
Cycle 1 (doxorubicin PK): 0 (postinfusion, Day 1); 0.5, 1, 2, 4, 8, 24, 48, 72, and 96 hours postinfusion (Days 1-5); preinfusion (Day 10)
Cycle 1 (olaratumab PK): preinfusion (Day 1); 0 (postinfusion, Day 10); 1, 4, 24, 48, 72, and 96 hours postinfusion (Days 10-14)
Cycle 2 (doxorubicin PK): 0 (postinfusion, Day 1); 0.5, 1, 2, 4, 8, 24, 48, 72, and 96 hours postinfusion (Days 1-5)
Cycle 2 (olaratumab PK): preinfusion (Day 1); 0 (postinfusion, Day 1); 1, 4, 24, 48, 72, and 96 hours postinfusion (Days 1-5); preinfusion (Day 8); 0 (postinfusion, Day 8); 1 and 4 hours postinfusion (Day 8)

Abbreviations: DDI, drug-drug interaction; DOXO, doxorubicin; PK, pharmacokinetic(s).

Supplemental Table 1. Estimated Pharmacokinetic Parameters

Parameter	Description
$\%AUC(t_{last}\text{-}\infty)$*	Fraction of area under the concentration versus time curve from zero to infinity (0-∞) extrapolated.
$AUC(0\text{-}\infty)$*	Area under the concentration versus time curve from zero to infinity.
$AUC(0\text{-}t_{last})$*	AUC from time zero to the last time point with a measurable concentration.
$AUC_{0\text{-}168h}$#	Area under the concentration versus time curve from 0 to 168 hours.
$AUC_{0\text{-}288h}$#	Area under the concentration versus time curve from 0 to 288 hours.
$AUC_{0\text{-}336h}$#	Area under the concentration versus time curve from 0 to 336 hours.
CL	Apparent total body clearance of drug calculated after IV administration.
C_{max}	Maximum observed drug concentration.
t_{max}	Time of maximum observed drug concentration.
$t_{1/2}$	Half-life of terminal elimination rate constant.
V_z	Volume of distribution during terminal phase.
V_{ss}	Volume of distribution at steady state apparent volume of distribution at steady state after IV administration.

*Parameter estimated for doxorubicin only.
#Parameter estimated for olaratumab only.

Supplemental Table 2. Adverse Events

	Part A - 15 mg/kg Olaratumab + 75 mg/m^2 Doxorubicin (N = 25)		Part B - 20 mg/kg Olaratumab + 75 mg/m^2 Doxorubicin (N = 24)	
	Any Grade[a]	Grade ≥3[a]	Any Grade[a]	Grade ≥3[a]
**Treatment-Emergent Adverse Events (n, % of Patients with Adverse Events; MedDRA Preferred Term)[b]**				
Fatigue[c]	14 (56.0)	1 (4.0)	19 (79.2)	0
Nausea	14 (56.0)	1 (4.0)	19 (79.2)	0
Musculoskeletal pain[d]	10 (40.0)	0	14 (58.3)	0
Mucositis[e]	9 (36.0)	1 (4.0)	12 (50.0)	3 (12.5)
Constipation	9 (36.0)	0	12 (50.0)	0
Anaemia	11 (44.0)	3 (12.0)	8 (33.3)	5 (20.8)
Neutropenia[f]	10 (40.0)	7 (28.0)	9 (37.5)	7 (29.2)
Diarrhoea	6 (24.0)	1 (4.0)	12 (50.0)	0
Cough	9 (36.0)	0	8 (33.3)	0
Alopecia	7 (28.0)	0	10 (41.7)	0
Decreased appetite	7 (28.0)	0	8 (33.3)	0
Vomiting	6 (24.0)	1 (4.0)	8 (33.3)	0
Thrombocytopenia[g]	9 (36.0)	5 (20.0)	4 (16.7)	2 (8.3)
Leukopenia[h]	8 (32.0)	6 (24.0)	4 (16.7)	3 (12.5)
Dizziness	4 (16.0)	0	7 (29.2)	0
Dysgeusia	4 (16.0)	0	8 (33.3)	0
Headache	6 (24.0)	0	4 (16.7)	0
Dyspnoea	5 (20.0)	2 (8.0)	5 (20.8)	1 (4.2)
Rash[i]	5 (20.0)	0	4 (16.7)	0
Abdominal pain[j]	3 (12.0)	0	6 (25.0)	0
Gastrooesophageal reflux disease	5 (20.0)	0	3 (12.5)	0
Pyrexia	4 (16.0)	1 (4.0)	4 (16.7)	0
Dry mouth	0	0	7 (29.2)	0
Oedema peripheral	0	0	5 (20.8)	0
Upper respiratory tract infection	0	0	5 (20.8)	0
Lymphopenia[k]	4 (16.0)	3 (12.0)	0	0
**Patients with any TEAE**	25 (100.00)	0	24 (100.00)	0
**Patients with adverse events leading to discontinuation from the study**				
Neutropenia	1 (4.0)	1 (4.0)	0	0
Febrile neutropenia	1 (4.0)	1 (4.0)	0	0
Hypersensitivity	1 (4.0)	1 (4.0)	0	0
**Patients with serious adverse events**	9 (36.0%)	0	9 (37.5%)	0

	Part A - 15 mg/kg Olaratumab + 75 mg/m^2 Doxorubicin (N = 25)		Part B - 20 mg/kg Olaratumab + 75 mg/m^2 Doxorubicin (N = 24)	
	Any Grade[a]	Grade ≥3[a]	Any Grade[a]	Grade ≥3[a]
Febrile neutropenia	1 (4.0%)	1 (4.0%)	3 (12.5%)	3 (12.5%)
Tumour haemorrhage	1 (4.0%)	1 (4.0%)	2 (8.3%)	2 (8.3%)
Neutropenia	2 (8.0%)	2 (8.0%)	1 (4.2%)	1 (4.2%)
Thrombocytopenia	2 (8.0%)	2 (8.0%)	1 (4.2%)	1 (4.2%)
Anaemia	1 (4.0%)	1 (4.0%)	1 (4.2%)	1 (4.2%)
Deep vein thrombosis	1 (4.0%)	1 (4.0%)	1 (4.2%)	1 (4.2%)
Pyrexia	2 (8.0%)	1 (4.0%)	0	0
**Adverse Events of Special Interest**				
Infusion-related reactions[l]	2 (8.0)	1 (4.0)	0	0
Cardiac arrhythmias[m]	2 (8.0)	0	4 (16.7)	1 (4.2)
Cardiac dysfunction[n]	9 (36.0)	1 (4.0)	6 (25.0)	0
Cardiac dysfunction (excluding oedema and peripheral oedema)	5 (20.0)	1 (4.0)	0	0

Abbreviations: MedDRA, Medical Dictionary for Regulatory Activities; N, number of patients studied; TEAE, treatment-emergent adverse event.

[a]Adverse events and clinical laboratory toxicity were graded according to the National Cancer Institute Common Terminology Criteria for Adverse Events (version 4.0). Any TEAEs with grade missing were counted as "Any Grade." At each level of summarization, a patient is counted once according to the TEAE with worst grade.

[b]The adverse events listed here were reported in at least 15% of patients. These included individual preferred terms from the Medical Dictionary for Regulatory Activities ([MedDRA] version 17.0) and specific consolidated terms combining clinically synonymous MedDRA preferred terms.

[c]Consolidated term comprising fatigue and asthenia

[d]Consolidated term comprising arthralgia, back pain, bone pain, flank pain, muscle spasms, musculoskeletal chest pain, musculoskeletal pain, myalgia, neck pain, and pain in extremity

[e]Consolidated term comprising mucosal inflammation, oropharyngeal pain, and stomatitis

[f]Consolidated term comprising neutropenia and neutrophil count decreased

[g]Consolidated term comprising platelet count decreased and thrombocytopenia

[h]Consolidated term comprising leukopenia and white blood cell count decreased

[i]Consolidated term comprising dermatitis, rash, rash macular, and rash pruritic

[j]Consolidated term comprising abdominal pain and abdominal pain upper

[k]Consolidated term comprising lymphopenia and lymphocyte count decreased

[l]Consolidated term comprising hypersensitivity and Infusion-related reaction

[m]Consolidated term comprising palpitations, syncope, arrhythmia, and sinus tachycardia

[n]Consolidated term comprising oedema, ejection fraction decreased, oedema peripheral, cardiomegaly, left ventricular dysfunction, and ventricular dysfunction
